# Interconception care in Australian general practice: a qualitative study

**DOI:** 10.3399/BJGP.2022.0624

**Published:** 2023-10-31

**Authors:** Sharon James, Cathy Watson, Elodie Bernard, Greasha K Rathnasekara, Danielle Mazza

**Affiliations:** National Health and Medical Research Council Centre of Research Excellence, School of Public Health and Preventive Medicine, Monash University, Victoria; Head, Department of General Practice, Monash University, Victoria.; Centre for Epidemiology and Biostatistics, Melbourne School of Population and Global Health, University of Melbourne, Victoria.; National Health and Medical Research Council Centre of Research Excellence, School of Public Health and Preventive Medicine, Monash University, Victoria; Head, Department of General Practice, Monash University, Victoria.; National Health and Medical Research Council Centre of Research Excellence, School of Public Health and Preventive Medicine, Monash University, Victoria; Head, Department of General Practice, Monash University, Victoria.; National Health and Medical Research Council Centre of Research Excellence, School of Public Health and Preventive Medicine, Monash University, Victoria; Head, Department of General Practice, Monash University, Victoria.

**Keywords:** family practice, general practice, postpartum, preconception care, primary health care, women

## Abstract

**Background:**

GPs provide care for women across the lifespan. This care currently includes preconception and postpartum phases of a woman’s life. Interconception care (ICC) addresses women’s health issues between pregnancies that then have impact on maternal and infant outcomes, such as lifestyle and biomedical risks, interpregnancy intervals, and contraception provision. However, ICC in general practice is not well established.

**Aim:**

To explore GP perspectives about ICC.

**Design and setting:**

Qualitative interviews were undertaken with GPs between May and July 2018.

**Method:**

Eighteen GPs were purposively recruited from South–Eastern Australia. Audiorecorded semi- structured interviews were transcribed verbatim and analysed thematically using the Framework Method.

**Results:**

Most participants were unfamiliar with the concept of ICC. Delivery was mainly opportunistic, depending on the woman’s presenting need. Rather than a distinct and required intervention, participants conceptualised components of ICC as forming part of routine practice. GPs described many challenges including lack of clarity about recommended ICC content and timing, lack of engagement and perceived value from mothers, and time constraints during consultations. Facilitators included care continuity and the availability of patient education material.

**Conclusion:**

Findings indicate that ICC is not a familiar concept for GPs, who feel that they have limited capacity to deliver such care. Further research to evaluate patient perspectives and potential models of care is required before ICC improvements can be developed, trialled, and evaluated. These models could include the colocation of multidisciplinary services and services in combination with well-child visits.

## INTRODUCTION

Both the occurrence and recurrence of poor pregnancy and birth outcomes are linked to a woman’s health status prior to conception.^1^ Interconception care (ICC) seeks to optimise the health of women who already have children, particularly those who have experienced a poor fetal or maternal outcome in the previous pregnancy. There is overlap between preconception, postpartum, and ICC care,^2^ but what is unique to ICC is optimising health for the next pregnancy with the knowledge of previous pregnancy outcomes.^3^ This includes optimising the management of any chronic health conditions, counselling regarding ideal interpregnancy interval, contraception provision, as well as identifying and providing advice about modifiable lifestyle and biomedical risk factors to prevent health risks for the mother and future pregnancies (Box 1).^3^

**Box 1.** Modifiable risk factor prevention during interconception

Biomedical risks are conditions that impact on a person’s risk of developing chronic illnesses and include high blood pressure, impaired fasting glucose, and dyslipidaemia.^21^ These risks are often influenced by lifestyle, such as smoking, nutrition, alcohol intake, and physical activity. 21 Biomedical risks predispose women to poor reproductive outcomes and poorer health across the lifespan,^22^ where having a chronic illness is associated with a higher rate of maternal and child morbidity and mortality. 23 For example:		
**Lifestyle risk**	**Potential impact on mother**	**Potential impact on child**
Smoking	Increases the risk of premature death, asthma, cardiovascular disease, and cancers^24^	Smoking during pregnancy can contribute to low birth weight, preterm birth, and ongoing impacts including asthma and obesity^25^
Nutrition and physical activity	A higher body mass index is a significant risk factor for the recurrence of gestational diabetes mellitus, increasing maternal pre-eclampsia risk^26^^,^^27^	Maternal undernourishment and underweight are risk factors for preterm birth,^28^ one of the leading causes of preterm mortality worldwide.^29^ Gestational diabetes increases the risk of neonatal hypoglycaemia^27^
Alcohol	The long-term effects of alcohol consumption on adults include increased risks of breast, liver, digestive, and colorectal cancers^30^	Maternal alcohol consumption increases risks such as preterm birth, low birth weight, and fetal alcohol spectrum disorder^31^^,^^32^

Existing Australian guidelines focus on antenatal and preconception preventive care, but not ICC.^4^^–^6 A lack of familiarity with ICC as a concept is a barrier to its implementation.^7^ Differing terminology used to describe ICC, including preconception care between pregnancies, internatal care, and interpregnancy care,^8^ adds complexity. While antenatal and postpartum care remains integral to optimising birthing and postnatal outcomes, targeting preventive care during these consultations may be too late to positively impact ongoing maternal and infant health. For example, half (121 million) of all pregnancies each year are unintended^9^ and yet planning interpregnancy intervals to be 12 to 24 months is optimal for perinatal outcomes.^10^ In addition, only 60% of women attend a primary care consultation in the first 12 months postpartum^11^ and these consultations tend to focus on more immediate issues such as breastfeeding challenges.^12^

There are many medical, lifestyle, psychological, and social risks during the interconception period that are preventable and amenable to care in general practice. Existing services in Australia, such as family planning clinics and community health centres, provide aspects of ICC, such as contraception provision and diabetes education. However, many GPs also provide shared antenatal care with public hospitals or birth centres and, importantly, offer continuity of care after birth and beyond.^13^ For example, ICC uptake is enabled where the usual place of care is easily accessible and there is a relationship with a doctor or nurse.^14^^,^^15^ While existing international empirical primary healthcare literature recognises interconception as a distinct life stage, it primarily comprises consumer and health professional surveys or inductively analysed data about ICC implementation facilitators, barriers, feasibility, and acceptability.^14^^–^20 This study seeks to fill a gap in this research by interviewing Australian GPs about ICC.

**Table:** How this fits in

Interconception care (ICC) provides an opportunity to address risk factors contributing to poor pregnancy outcomes. However, GP perceptions on providing ICC are not well established. ICC is not a familiar concept for GPs, it is delivered opportunistically, and there is lack of clarity as to what ICC should consist of. GPs also feel there is lack of engagement and perceived value by women.

## METHOD

### Aim and research questions

This study aimed to explore Australian GP perceptions, knowledge, and reported practices regarding ICC. The research questions were:

1. What do GPs know and understand about the interconception period and ICC?
2. What is GPs’ perceived role in providing ICC? and
3. What are the barriers and facilitators to ICC delivery in general practice?

### Design, participants, and recruitment

This is a qualitative study conducted in South–Eastern Australia, and the COnsolidated criteria for REporting Qualitative (COREQ) research checklist was used to structure the conduct and reporting of the study.^33^

GPs, including two pilot interviewees, were purposively recruited by mail between May and July 2018 using the Monash practice-based Research Network (MonREN) database in Melbourne, Australia. To ensure GP participants were likely to have exposure to caring for women during the interconception period, only those who worked ≥4 clinical sessions per week could be part of the study. GP trainees and GPs who did not routinely care for women of reproductive age (15 to 45 years) were excluded from the study.

### Data collection

Prior to interview, prospective participants were given an explanatory statement that included details about who was conducting the research and the study’s aims and objectives. None of those who responded refused to participate. Participant consent and rapport building was undertaken prior to interview. To meet the individual needs of GPs, one- on-one semi- structured interviews were undertaken by phone from a private office by one of the researchers, a female medical Honours student with an interest in women’s health, using an interview guide (Box 2). To support conceptual discussion about ICC, interviewees were given a brief definition of this terminology at the start of the interview as the ‘care of women between pregnancies’. Field notes were undertaken after each interview to describe the researcher’s interview experience.

**Box 2.** Examples of interview questions

What is your understanding of interconception care? How does this translate into your everyday practice? What do you think are the major risk factors affecting women between their pregnancies? How important do you feel interconception care is? That is, what difference (if any) does it make? Tell the researcher about a particular patient in your practice for whom you have provided interconception care and how it benefited them. Whose role is it to undertake interconception care? The hospital, the GP, the practice nurse, or someone else? Do you think women of childbearing age in your practice understand the concept of interconception care? Do women in your practice value optimising their health between pregnancies for a future pregnancy? Is this different if a woman has experienced a poor previous pregnancy outcome? What do you perceive as barriers to the systematic delivery of interconception care? What currently helps women to receive interconception care?

The interview schedule was informed by a literature review, conducted by primary care academics with experience in women’s health and qualitative research methods. Interview piloting was undertaken with a trainee GP and two GPs. De-identified audio files were transcribed verbatim by a professional transcription company and imported into NVivo (version 11) for analysis. Participants received a $150 gift card on completion of the interview. Repeat interviews or the return of transcripts or findings to participants were not undertaken.

### Rigour

Lincoln and Guba’s criteria of credibility, transferability, dependability, confirmability, and authenticity can be applied to studies to establish trustworthiness.^34^ Credibility was established through field notes and peer/research team debriefing. Strategies to support transferability included the thick description and saturation of interview data. Prospective participants could contact the researchers to confirm details about the study and research team. Transferability was established through the recruitment of a representative sample of GPs and a review of the literature to confirm the findings. Methods used to enhance dependability included research team discussions about data interpretations, as well as auditable documentation. Finally, confirmability was established through analysis codebook development and the research team’s reflexivity.

### Data analysis

Ritchie and Spencer’s ‘Framework Method’ was used to inductively analyse data.^35^ The Framework Method consists of key stages in the research process, allowing researchers to thematically analyse data in five stages — familiarisation, coding, developing an analytical framework, charting data into a matrix, and finally interpretation.^35^ Familiarisation occurred by reading transcriptions and allocating descriptive codes relevant to the research question. After the initial codes were developed, these were grouped into emerging categories (see Supplementary Table S1), informing the working analytical framework or coding manual. This analytical framework was applied to the remaining transcripts and codes classified into relevant categories. Where relevant emergent information could not be classified into an existing category, these were coded and filed under ‘new codes’. New codes were then filed into suitable categories or formed new categories as part of an iterative process. Cross-checking of codes was done by two authors, one of whom was a female medical Honours research student, where three transcripts were discussed. Any discrepancies were discussed by two of the authors. Then, all the codes were simplified and sorted into categories and subcategories. Any redundant or repetitive codes were eliminated to give the final coding manual. Each interview was then reviewed for all codes associated with a particular theme according to the categories of coding. A summary of each participant’s codes for individual themes was charted into the analytical matrix, and this process was repeated for each major category and each participant to allow a cross-case comparison and analysis. Data saturation occurred at 18 interviews, when no new themes emerged and no further interviews were undertaken. Relevant quotes were indexed in the matrix for interpretation.

## RESULTS

Most participants were female (*n* = 17/18), with a mean age of 40 years, and were not a shared antenatal care provider (*n* = 13/18) (Table 1). Five participants were shared care providers and most participants were generally unfamiliar with ICC as a concept regardless of experience level. Audiorecorded telephone interviews were 30–60 min (mean 39.5 min) in duration.

**Table 1.** Demographic characteristics of participating GPs (*n* = 18)

**Characteristic**	***n* (*%*)**
**Sex**	
Female	17 (94.4)
Male	1 (5.6)

**Age, years**	
25–34	7 (38.9)
35–44	4 (22.2)
45–54	7 (38.9)

**Shared maternity care provider**	
Yes	5 (27.8)
No	13 (72.2)

Three major themes were found from the interviews: lack of clarity as to what ICC entailed; opportunistic delivery; and the challenges faced by GPs in engaging mothers in ICC.

### ICC … *‘it’s not a definitive thing’*

While participants had varying descriptions of how ICC translated into practice, most understood the meaning but conceptualised it as preconception, pregnancy, or early postpartum care rather than health optimisation, or felt that the term was not typically used in general practice:

*‘Providing care from pre-pregnancy planning, through when they are pregnant, and then facilitating them until they either get onto sort of specialist care or through care via the hospital.’*

(GP15, female [F], aged 32 years)

*‘It’s not labelled as such, but you often get the preconception term, but you don’t really hear the interconception term so much.’*

(GP10, male, aged 34 years)

Most participants, however, did not conceptualise the interconception period as a distinct form of care to be delivered at specific points during a woman’s reproductive life. Rather, many viewed ICC as routine care for women of childbearing age:

*‘I actually don’t see it as a specific thing, I see it as a continuum, so I don’t actually need to make it a specialty because it’s just what we do … But it’s got to incorporate a whole lot of different aspects of general practice at the same time.’*

(GP3, F, aged 53 years)

*‘Not really conceptualising it as an actual need, you know there’s routine antenatal visits, routine six-week postnatal check-up, there’s the timed visits for immunisations with the bub but this doesn’t really exist as an entity that we plan for or schedule.’*

(GP1, F, aged 54 years)

Regardless of how participants conceptualised ICC, all felt ICC to have *‘the potential to make a huge difference’* (GP13, F, aged 47 years) in optimising future maternal and infant outcomes:

*‘I feel it’s very important, because we have to have … a healthy mum, to have a healthy, well baby, and any issues and complications are going to affect that baby in utero … for its whole life, so you’re going to have decades of consequences if there is poor interconception care.’*

(GP8, F, aged 34 years)

### GPs demonstrate some best practice … but it’s opportunistic

#### Opportunistic delivery

Participants felt they had a major role in delivering ICC because of their accessibility and knowledge of the individual woman’s health issues and risk factors. There are occasions to deliver care during the interconception period; however, *‘… you might only be able to provide this kind of interconception care on an opportunistic basis’* (GP14, F, aged 29 years).

Much of their interconception contact with women occurred at the initial 6-week visit ‘*when the baby comes in for their immunisations’* (GP9, F, aged 32 years). This was dedicated to the review of physical healing from delivery, mental health, coping with a newborn, breastfeeding, and follow-up of any pregnancy complications, rather than focused on optimising health for a subsequent pregnancy:

*‘At the six-week check I think that’s a great starting point … actually, at the moment, I do it opportunistically depending on what they’re coming in for, but sometimes just mentioning to come in before they fall pregnant or a few months before they fall pregnant or plan to start pregnancy but then practically I find that women don’t end up doing it.’*

(GP11, F, aged 44 years)

Some participants expressed concern that patients were not receptive to discussing future pregnancies and safe pregnancy spacing early in the postpartum period, preferring to leave that discussion to a later appointment or *‘some point when they’re not so tired’* (GP1, F, aged 54 years). However, other participants prioritised eliciting their patients’ future pregnancy plans but didn’t seem to focus on what was an optimal pregnancy interval. In the main, oral contraception was commonly prescribed for women who wanted a shorter pregnancy interval, while long- acting reversible contraceptives were offered for women who had completed their families or desired longer pregnancy spacing:

*‘Depending on what their plans are you will tailor the contraception advice … So, I always ask them that at that stage and I always tell them, before you start trying to get pregnant again, make sure you come in at least a few months before just to have some pre-pregnancy counselling and tests.’*

(GP6, F, aged 45 years)

#### GP knowledge about ICC

 Participants’ knowledge about the likelihood of recurrent poor birth outcomes varied. Issues discussed included the potential for fetal growth problems and preterm labour, prevention, and follow-up of gestational diabetes mellitus, significant weight gain, gestational hypertension, pre-eclampsia, pelvic floor dysfunction, and postnatal depression:

*‘If the mother’s had severe post-natal depression, then it’s something that I just outright flag and say well you’ve had this with the first one, you might have similar feelings for the second one, and if you want to come back in X amount of weeks or months, then we’re always here or if she’s already meeting with psychologists, to make sure you encourage her to keep up with the psychology … ’*

(GP18, F, aged 45 years)

*‘So, usually I see a lot of mums who end up getting gestational diabetes and one thing I usually try to say to them when we find out that they’re pregnant or in subsequent pregnancies is to discuss about weight gain during pregnancy as well and also in the sort of postpartum period … ’*

(GP9, F, aged 32 years)

For women at risk of recurrent poor outcomes secondary to lifestyle risks, participants described offering more frequent postpartum follow-ups, more lifestyle risk reduction advice, and early antenatal care access in future pregnancies. Where lifestyle counselling between pregnancies did occur, participants reported this as part of discussion about early folic acid use, weight optimisation, management of chronic illnesses, alcohol and smoking cessation, updating vaccinations, ensuring women are using safe medications, and performing preconception blood tests:

*‘In an ideal world it would be obviously optimising weight, having healthy diet, optimising fitness, and having regular exercise. Making sure that they, whenever they plan to, if it’s, you know, further down the line, then at least be on their prenatal vitamins for at least three months before starting. Stop smoking if they’re smoking, reduce alcohol consumption, reduce caffeine intake.’*

(GP13, F, aged 47 years)

There was some concern about offering counselling about sensitive topics, such as weight reduction, and the potential for damaging their rapport and stigmatising women if this was discussed at *‘every single visit’* (GP2, F, aged 34 years). However, participants believed that a trusting relationship and familiarity with the woman’s medical history and social context facilitated these opportunistic discussions:

*‘We see the children, we see the mothers most often, often quite frequently when the family’s young. So, there is that opportunity to monitor things. But you have to, I suppose, be receptive and be prepared to go down that path to be able to provide that care to identify the issues.’*

(GP16, F, aged 44 years)

#### ICC timing

When GPs were asked about the ideal time to discuss health optimisation for a subsequent pregnancy, some gave specific time frames of 6 to 12 months postpartum, or 3 months prior to conception. Yet, in practice, participants described two distinct opportunities to provide ICC. First, ICC could occur during general women’s health visits, such as contraceptive pill script refills, cervical screening tests, and concerns regarding menstruation:

*‘I say, well, women’s check-up today, we’re going to talk about contraception, and smoking, alcohol, exercise, and just work that in, and say this is a routine thing and I do it with every woman who comes to me for the cervical screen.’*

(GP8, F, aged 34 years)

Second, participants described ICC as occurring when a woman presents with their baby, generally in the first year postpartum, to ask mothers about their own health and wellbeing, reminding them about necessary tests and providing lifestyle advice. One GP discussed scheduling reviews of a mother’s health progress during timed immunisation visits for the infant:

*‘I try to line them up with the immunisation schedule of the baby so it’s much easier for them to come at two, four, and six months so they don’t have to take extra time or special effort.’*

(GP7, F, aged 40 years)

### GPs face many challenges engaging mothers in ICC

Participants felt that most women, regardless of parity, do not prioritise health optimisation for a subsequent pregnancy, and the *‘majority of patients turn up already pregnant’* (GP12, F, aged 32 years).

*‘I would say less than fifty per cent have a consultation when it’s their second pregnancy. They nearly all do with their first pregnancy but not many do when it’s their second pregnancy … just because they have done it before they think they know everything, so I do emphasise to them that I think it’s important to come in for a check- up before they start trying to get pregnant again.’*

(GP5, F, aged 35 years)

Some felt that, while most mothers still value personal health optimisation for a subsequent pregnancy, they can experience a lack of support and have numerous competing interests, including family and employment responsibilities:

*‘I think the intention is there regardless, to look after their own health, whether it’s first- time or returning mums. But I think … mums where there’s one or more children around, the focus shifts and it becomes much more about the child and much less about themselves.’*

(GP16, F, aged 44 years)

*‘… often what will happen is the mum will have an appointment for me and she’ll end up giving it to the baby because the baby’s got a fever … ’*

(GP1, F, aged 54 years)

A few participants stated that women who had experienced a complicated previous pregnancy valued ICC and *‘have been more proactive’* (GP9, F, aged 32 years) to prevent recurrence of poor pregnancy outcomes *‘especially when things haven’t gone quite as well as they wanted’* (GP14, F, aged 29 years).

Participants also believed time constraints to be another barrier to ICC. A standard consultation time was thought to be too short to address both the patient’s presenting complaint and ICC:

*‘… most of the time you don’t have adequate time to provide it … you can encourage someone to come back … but then it also means that they do have to come back and give you that amount of time to be able to do it.’*

(GP14, F, aged 29 years)

Participants stressed the need to *‘raise awareness that women should be seeking medical opinions before they … fall pregnant’* (GP17, F, aged 53 years) and considered practice-based initiatives, including patient education materials such as posters and brochures in the waiting room, apps, and social media:

*‘… we send … a monthly newsletter … and we’ve got a TV in the waiting room. So, I guess we could always put information in those … or on our Facebook page … to try and raise awareness of interconception care, and encourage patients to see their doctor … ’*

(GP12, F, aged 32 years)

There was an identified need for additional specific funding of general practice to support ICC, allowing GPs to spend more time with patients. In addition, funding for mothers to access allied health practitioners for modifiable risk factors was suggested, similar to funding approaches used in chronic disease management:

*‘You may need to involve a psychologist; you may need to involve a dietitian; you may need to involve a diabetes educator; we need to use our practice nurse to facilitate things.’*

(GP5, F, aged 35 years)

*‘My answer to that is the same as improving anything which is increasing the Medicare rebates (funding for general practice) because I think they’re very, very poor and they don’t allow the time with patients and they don’t allow for good preventative care.’*

(GP2, F, aged 34 years)

## DISCUSSION

### Summary

Although ICC is commonly referred to in the international literature, GPs are not familiar with the concept and described a lack of patient engagement in this care. While raising awareness is one way to enhance uptake and delivery of ICC services, the opportunistic nature of current ICC delivery is reflective of the appointment structure and funding model for general practice services. This has implications for the ongoing engagement and health optimisation of the mother as well as the prevention of potential adverse pregnancy outcomes.

### Strengths and limitations

The strength of this study relates to the qualitative approach, adding insight into the limited knowledge about ICC and showing GPs’ understanding, knowledge, and practices of ICC. The study aimed to recruit GPs who were in regular contact with women of childbearing age and, in doing so, the knowledge and perspectives of ICC may not be generalisable to all GPs. Similarly, the study attracted predominantly female and metropolitan GPs; however, this may be reflective of the GP workforce profile^36^^,^^37^ and patient preference for ICC services. Further research should obtain the views of male and rural GPs as well as other stakeholders in ICC.

### Comparison with existing literature

ICC was largely opportunistic in nature as part of postpartum care or infant immunisations. Where ICC was targeted, the types of patient visits were ‘problem focused’, such as menstrual issues and oral contraceptive prescriptions. However, this approach does not address all aspects of ICC. Lessons from ICC literature identify that tailoring interventions to the health and social needs of women is needed, and support for embedding this into routine care is required.^15^ The ability of services to meet the needs of women requiring ICC can be problematic where women juggle caring and work demands,^38^ where existing models of care are fragmented between services,^39^ or where a subsequent appointment is required. For example, the delivery of efficacious forms of contraception care, such as long-acting reversible contraception, often require a return appointment.^40^ The integration of ICC into other appointments, such as for the child, is one solution proposed by international ICC experts,^16^^,^^17^^,^^19^^,^^20^^,^^41^ provided that mechanisms supporting this care include adequate provider remuneration and guidelines.^16^

Health optimisation through the prevention and management of lifestyle risk, biomedical risk factors, and adverse pregnancy outcomes were not discussed by participants, unless there was concern about a recurrence of poor pregnancy outcome. While also applicable to ICC more broadly, the integration of lifestyle risk reduction in consultations occurs where it is perceived as relevant and integrated into routine care.^42^ Similar to other literature about GP-led lifestyle interventions, the importance of addressing lifestyle behaviours was acknowledged; despite this, these discussions were described as being undertaken in an *ad-hoc* way.^43^ The potential impact of lifestyle and biomedical risks increase with maternal age and parity.^44^ Obesity, for example, can increase the risk of comorbidities during pregnancy, as well as offspring epigenetic and the mother’s ongoing chronic disease risk.^45^ Preconception and antenatal preparation for lifestyle risk reduction can be too late to see the outcomes needed for a subsequent pregnancy.^46^ To support the woman’s health and allow adequate time in the reduction of lifestyle risks, including dietary intake and inadequate physical activity, months or years prior to conception may need to be allocated^1^ and therefore discussed as part of routine ICC.

Participants perceived issues engaging women in ICC. This is consistent with other literature indicating that, while 74% plan their pregnancies, less than half seek out health professional advice.^47^^,^^48^ The disconnect between women planning a pregnancy and getting advice could be because of cost, patient time, accessibility of care, or the number of GPs providing this service. In addition, women’s engagement in primary care services postpartum is high but inconsistent.^49^ This may be due to the issues being faced by women at that time such as recovery, poor sleep patterns, adjustment, and mental health issues. Subsequent visits to the GP also focus on the baby rather than the mother, further impacting this engagement.

Participating GPs perceived that women do not prioritise their own health for a subsequent pregnancy unless there had been a complication. While participants also recognised that there are many competing priorities during the interconception period, there is evidence to suggest that multiparous women are more knowledgeable or relaxed about subsequent pregnancies.^47^ Engagement in care is also impacted by receptiveness to information about preconception health,^50^ costs, and having an ongoing relationship with a practice, GP, or nurse.^14^ To address increased risks from modifiable risk factors, age, and parity, health optimisation should be discussed across the lifespan, not just before pregnancy. Approaches that target those who may benefit from ICC could involve the colocation of publicly funded and family-friendly multidisciplinary services that could include nurses, midwives, diabetes educators, dietitians, and women’s health physiotherapists. In addition, the allocation of funded time for ICC in combination with well-child visits, such as coinciding with immunisation schedule presentations, is another possible solution.

### Implications for research and practice

Most GPs do not currently view interconception as a distinct stage in a woman’s reproductive lifespan to promote health optimisation for a subsequent planned pregnancy. Funded opportunities and guidelines for care could facilitate routine incorporation of ICC into clinical practice. This includes outlining clinical content areas that fall under the banner of ICC, as well as the need for this care to occur months and years prior to conception. Research needs to elicit the views of both female and male, urban and rural GPs, and other key stakeholders, such as nurses and specialists, involved in ICC. In addition, the testing of new approaches that overcome barriers to delivering ICC is required. Publicly funded and family-friendly multidisciplinary care that considers when women are likely to present to general practice during interconception, such as well-child visits, are key opportunities for service provision.
